# Hypoxia Aggravates Inactivity-Related Muscle Wasting

**DOI:** 10.3389/fphys.2018.00494

**Published:** 2018-05-15

**Authors:** Tadej Debevec, Bergita Ganse, Uwe Mittag, Ola Eiken, Igor B. Mekjavic, Jörn Rittweger

**Affiliations:** ^1^Faculty of Sport, University of Ljubljana, Ljubljana, Slovenia; ^2^Department of Automation, Biocybernetics and Robotics, Jožef Stefan Institute, Ljubljana, Slovenia; ^3^Department of Orthopaedic Trauma, RWTH Aachen University Hospital, Aachen, Germany; ^4^Institute of Aerospace Medicine, German Aerospace Center, Cologne, Germany; ^5^Department of Environmental Physiology, Swedish Aerospace Physiology Centre, KTH Royal Institute of Technology, Stockholm, Sweden; ^6^Department of Biomedical Physiology and Kinesiology, Simon Fraser University, Burnaby, BC, Canada; ^7^Department of Pediatrics and Adolescent Medicine, University of Cologne, Cologne, Germany

**Keywords:** muscle loss, bed rest, hypoxemia, vastus lateralis, fiber type

## Abstract

Poor musculoskeletal state is commonly observed in numerous clinical populations such as chronic obstructive pulmonary disease (COPD) and heart failure patients. It, however, remains unresolved whether systemic hypoxemia, typically associated with such clinical conditions, directly contributes to muscle deterioration. We aimed to experimentally elucidate the effects of systemic environmental hypoxia upon inactivity-related muscle wasting. For this purpose, fourteen healthy, male participants underwent three 21-day long interventions in a randomized, cross-over designed manner: (i) bed rest in normoxia (NBR; P_i_O_2_ = 133.1 ± 0.3 mmHg), (ii) bed rest in normobaric hypoxia (HBR; P_i_O_2_ = 90.0 ± 0.4 mmHg) and ambulatory confinement in normobaric hypoxia (HAmb; P_i_O_2_ = 90.0 ± 0.4 mmHg). Peripheral quantitative computed tomography and vastus lateralis muscle biopsies were performed before and after the interventions to obtain thigh and calf muscle cross-sectional areas and muscle fiber phenotype changes, respectively. A significant reduction of thigh muscle size following NBR (-6.9%, SE 0.8%; *P* < 0.001) was further aggravated following HBR (-9.7%, SE 1.2%; *P* = 0.027). Bed rest-induced muscle wasting in the calf was, by contrast, not exacerbated by hypoxic conditions (*P* = 0.47). Reductions in both thigh (-2.7%, SE 1.1%, *P* = 0.017) and calf (-3.3%, SE 0.7%, *P* < 0.001) muscle size were noted following HAmb. A significant and comparable increase in type 2× fiber percentage of the vastus lateralis muscle was noted following both bed rest interventions (NBR = +3.1%, SE 2.6%, HBR = +3.9%, SE 2.7%, *P* < 0.05). Collectively, these data indicate that hypoxia can exacerbate inactivity-related muscle wasting in healthy active participants and moreover suggest that the combination of both, hypoxemia and lack of activity, as seen in COPD patients, might be particularly harmful for muscle tissue.

## Introduction

Poor functional and musculoskeletal state is commonly observed in numerous clinical populations. Muscle dysfunction and consequent muscle weakness are, for example, both prominent features of chronic obstructive pulmonary disease (COPD) (Maltais et al., 2014; Sillen et al., 2014) as well as heart failure (Harrington et al., 1997) patients and have moreover been shown to independently modulate mortality and morbidity (Marquis et al., 2002; Swallow et al., 2007; Patel et al., 2014). While physical inactivity is currently thought to be the key underlying cause of muscle wasting observed in COPD (Debigare et al., 2001; Maltais et al., 2014), systemic hypoxemia (Koechlin et al., 2005) could also contribute to muscle deterioration. Proper determination of the contribution of systemic hypoxia to muscle wasting is unfeasible in clinical populations. Therefore, prospective, mechanistic investigations in healthy individuals are warranted to provide key insights into the underlying factors.

Various levels of inactivity are known to promote skeletal muscle mass wasting in both health and disease (Narici and de Boer, 2011; Puthucheary et al., 2013). The detrimental effects of inactivity on muscle morphology in humans have been clearly established using various experimental approaches from single limb suspension (Berg et al., 1991) to bed rest (Pavy-Le Traon et al., 2007). The bed rest experimental model, initially established as an earth-based analog to explore the effects of space-travel related microgravity (Deitrick et al., 1948), is especially valuable as it provides a unique insight into the integrative physiological deconditioning in response to whole body unloading. Bed rest investigations that were performed over the last half-century clearly showed that whole body inactivity generally provokes muscle atrophy of the lower limbs at an initial rate of about 2–3% per week (Pavy-Le Traon et al., 2007; Narici and de Boer, 2011; Ploutz-Snyder, 2016). Thus, previous work suggests that a ∼7–12% reduction of the thigh and calf muscle mass can be expected following 3 weeks of bed rest-induced inactivity (Kawakami et al., 2000, 2001; Akima et al., 2001; Kubo et al., 2004). Noteworthy, whole body inactivity as short as a few days not only downregulates muscle volume but also promotes muscle phenotype changes from slow twitch (oxidative; type 1) to fast twitch (glycolytic; type 2) muscle fibers without fiber attrition (Brooks and Myburgh, 2014; Demangel et al., 2017).

In contrast to inactivity, the effects of systemic hypoxia on muscle mass modulation are less clear. While some studies in humans reported high altitude-related muscle mass reduction (Boutellier et al., 1983; Hoppeler et al., 1990; MacDougall et al., 1991) others did not (Green et al., 2000; Lundby et al., 2004; D’Hulst et al., 2016). Obviously, many factors such as dietary intake, water balance, sleep quality and/or environmental cold exposure might have contributed to discrepancies between study outcomes. Nevertheless, an independent and dose-dependent effect of hypoxia on muscle mass regulation has been established in numerous rodent studies (Bigard et al., 1996; Favier et al., 2010). The reported influence of hypoxia inducible factor-1 (HIF-1α), which is stabilized under hypoxic conditions, on enzymatic regulation of skeletal muscle mass via central molecular node mammalian target of rapamycin inhibition (mTOR) lends further support to this notion (Favier et al., 2015). Recent data also suggest differential sensitivity of oxidative and glycolytic muscles to hypoxia-induced muscle atrophy with type 2 muscle fibers being particularly sensitive to reduced O_2_ availability (de Theije et al., 2015). The hypoxic dose (i.e., duration and “intensity” of hypoxic stimuli) therefore seems to be one of the key factors of hypoxia-related muscle mass regulation. As noted in a recent view-point (D’Hulst and Deldicque, 2017) along with subsequent commentaries (Millet et al., 2017), the threshold dose above which noticeable muscle wasting occurs is difficult to determine. Importantly, our previous work in this area suggests that 10-days of continuous hypoxia exposure (simulated altitude of ∼4000 m) does not independently modulate whole-body composition (Debevec et al., 2014b,c). However, potential additive effects of prolonged hypoxia on inactivity-related muscle wasting have, to date, not yet been investigated.

We sought to experimentally examine the effects of systemic environmental hypoxia upon inactivity-related thigh and calf muscle wasting. For this purpose, fourteen healthy male participants underwent the following three 21-day long experimental interventions: (i) bed rest in normoxia to determine the independent effect of inactivity (ii) bed rest in hypoxia to determine the additive effects of hypoxia to inactivity and (iii) ambulatory confinement in hypoxia to determine the effects of hypoxia *per se*. We hypothesized that the addition of hypoxia to inactivity will further exacerbate muscle mass reduction (primary hypothesis) and moreover, that hypoxia combined with habitual activity levels will not significantly modulate muscle mass of the lower limbs (secondary hypothesis).

## Materials and Methods

The present work represents a sub-study of a large, international project funded by the European Union (Planetary Habitat Simulation – PlanHab) conducted at the Olympic Center in Planica, Slovenia that aimed to investigate physiological responses to simulated microgravity and normobaric hypoxia. Comprehensive methodological details of the PlanHab project (Debevec et al., 2014a) have already been published along with several physiological outcomes (Debevec et al., 2016; Keramidas et al., 2016a,b; Rittweger et al., 2016; Rullman et al., 2016; Simpson et al., 2016; Morrison et al., 2017; Sket et al., 2017a,b; Strewe et al., 2017, 2018; Šket et al., 2018; Stavrou et al., 2018a,b). A brief summary of the projects’ key methodological features is provided below, but we kindly refer the readers to the above-mentioned papers for specific details.

### Participants

Based on the extensive selection procedure and preliminary testing we recruited 14 healthy, male, near sea-level residents to take part in the study. Their baseline characteristics were: Mean age = 26.4 (SE 1.4) years, Body height = 179.6 (SE 1.4) cm, Body mass = 75.9 (SE 2.8) kg, Body fat percentage = 21 ± (SE 1.9) %, Body Mass Index = 23.5 (SE 0.8) kg⋅m^-2^, and Peak oxygen uptake = 44.3 (SE 1.9) mL⋅kg^-1^⋅min^-1^. The participants’ habitual physical activity was assessed using the general health questionnaire (GHQ). They provided written informed consent prior to the start of experimental procedures. Recruitment procedures along with inclusion and exclusion criteria base on the European Space Agency recommendations for bed rest protocols (Standardization of bed rest study conditions 1.5, 2009) are detailed elsewhere (Debevec et al., 2014a; Sket et al., 2017b).

### Study Outline

**FIGURE 1:**
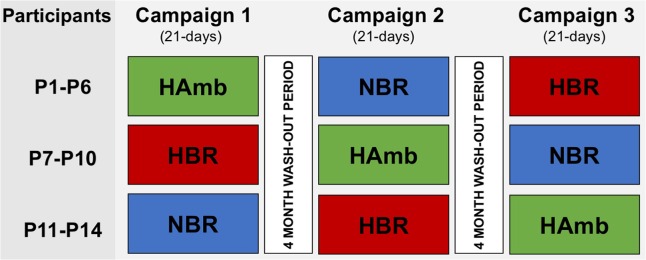
Cross-over designed allocation of the 14 participants (P1–P14) to the respective interventions (NBR, normoxic bed rest; HBR, hypoxic bed rest; HAmb, hypoxic ambulation) during the course of the project.

Participants underwent three 21-day interventions in a cross-over design over the course of 1 year with a 4-month wash-out period between interventions. They were randomized to the following three interventions, while each of them participated in each intervention once: (i) bed rest in normoxia (NBR; Fraction of ambient oxygen (FO_2_) = 0.209; Partial pressure of inspired oxygen (P_i_O_2_) = 133.1 ± 0.3); (ii) bed rest in normobaric hypoxia (HBR; FO_2_ = 0.141 ± 0.004; P_i_O_2_ = 90.0 ± 0.4; ∼4000 m), and (iii) confinement in normobaric hypoxia combined with habitual daily activity (HAmb FO_2_ = 0.141 ± 0.004; P_i_O_2_ = 90.0 ± 0.4). The allocation and randomization of the participants to each intervention over the course of the project is depicted in **Figure 1**. The present paper details an investigation into the effects of (in)activity and hypoxia on muscle wasting. For this purpose, peripheral quantitative computed tomography scans and vastus lateralis muscle biopsies were performed before and after the interventions to obtain thigh and calf muscle cross-sectional areas and muscle fiber phenotype changes, respectively. The project was approved by the National Committee for Medical Ethics of the Republic of Slovenia, conducted in accordance with the Declaration of Helsinki guidelines and registered at ClinicalTrials (NCT02637921).

### Bed Rest and Hypoxic Procedures

As detailed elsewhere (Debevec et al., 2014a), the participants were confined to strict horizontal (0°) inactivity throughout both bed rest interventions (NBR and HBR). They performed all activities (i.e., eating, reading, watching television) in the horizontal position, and were only allowed to change position from supine to lateral and prone. One pillow was permitted for head support. At least one shoulder had to touch the mattress at all times. Daily hygiene procedures were also performed in a horizontal position using a specific gurney. Bed rest protocol compliance was monitored throughout the intervention via constant research, medical staff supervision and closed-circuit television recordings. The participants were strictly forbidden to perform any other form of static or dynamic exercise.

During HAmb participants were encouraged to engage in their habitual routines but remained confined to the hypoxic area of the research facility throughout the intervention. They also performed two 30-min low-intensity exercise sessions daily (one in the morning and one in the afternoon) to mimic their normal daily physical activity. The exercise mode was alternated between stepping, cycling, and dancing to avoid monotony. Targeted exercise heart rate (HR, 123 ± 4 beats⋅min^-1^) was set based on heart rate at 50% peak power output obtained during the graded exercise tests performed at onset of the intervention. Continuous telemetry HR monitoring was used to ensure that participants were within the targeted zone during all exercise sessions.

Environmental conditions within the hypoxic facility were precisely controlled and remained stable throughout all interventions (ambient temperature = 24.4 ± 0.7°C; relative humidity = 53.5 ± 5.4% and ambient pressure = 684 ± 4 mm Hg). Hypoxia was generated via a vacuum pressure swing adsorption system (b-Cat, Tiel, the Netherlands). O_2_ and CO_2_ concentrations were monitored and regulated within each room at 15-min intervals. Participants wore portable O_2_ sensors (Rae PGM-1100, RAE Systems, CA, United States) for safety reasons at all times. Capillary oxyhemoglobin saturation (SpO_2_) was assessed each morning using a fingertip oximetry device (3100 WristOx, Nonin Medical, Plymouth, MN, United States). The 30-second averages of both values were used for subsequent analysis. To determine presence and severity of acute mountain sickness, the self-assessment part of the Lake Louise questionnaire was completed by participants every evening (Roach et al., 1993).

### Nutritional Strategy and Monitoring

The same dietary approach was employed during all three campaigns. As extensively detailed elsewhere, (Debevec et al., 2014a) intakes and dietary macronutrient composition were standardized throughout each intervention using a repeating meal plan (14-day menu; five meals per day). Targeted energy intakes were individually calculated using the Harris-Benedict equation resting metabolic rate equation (Hasson et al., 2011), accounting for activity levels of participants (i.e., whether ambulatory or bedridden). Dietary items were provided in weighed portions with the participants encouraged to consume all of the provided food. The amounts actually consumed were recorded and analyzed using a nutritional analysis program (Open Platform for Clinical Nutrition, Jožef Stefan Institute, Ljubljana, Slovenia).

### Peripheral Quantitative Computed Tomography

Muscle cross-sectional areas in the calf and thigh were assessed before (6th day before the start of each intervention), during (the 2nd, the 10th, and the 21st intervention day) and 14 days after cessation of the interventions using peripheral quantitative computed tomography (pQCT; XCT3000; Stratec Medizintechnik). As described previously (Rittweger et al., 2016) scout views in the frontal plane were performed on the tibio-talar joint and knee joint to identify distal and proximal tibia joint surfaces, and distal femur surfaces. Horizontal pQCT scans were obtained at 66% of the tibia length, and at 33% of the femur length (on both occasions the percentages were assessed from the distal ends of bones). Image analysis was performed using designated software (XCT3000 version 5.4). This software normalizes bone mineral density to density of fat tissue with density of muscle tissue amounting to about 70 mg⋅cm^-3^, and completely mineralized bone density about 1200 g⋅cm^-3^. Images were taken with a voxel size of 0.4 mm (parameter voxel size in the integrated software) in the transverse direction and 2.4 mm in the longitudinal (X-ray beam width). Regions of interest (ROI) were circulated around the outside of the muscles and within the subcutaneous tissue. Within ROIs, the total muscle area was determined using a 35 mg⋅cm^-3^ threshold (contour mode 1); representing a value in between the typical muscle (70 mg⋅cm^-3^) and fat (0 mg⋅cm^-3^) tissue density. Total areas of bones (fibula, tibia, and femur) were assessed using a threshold of 710 mg⋅cm^-3^ at contour mode 1. In order to obtain muscle cross-sectional areas only, the determined bone areas were subtracted from total muscle areas.

### Muscle Biopsies and Biochemical Procedures

Details of the muscle biopsy procedure performed in the present study were published elsewhere (Rullman et al., 2016). Briefly, muscle biopsy samples were obtained in the morning after ≥10 h of fasting from the vastus lateralis muscle before and at the end of each intervention (6 days before the start of each intervention and at the 21st day of the intervention). While the baseline biopsies were obtained from all participants in normoxic conditions, the post-intervention biopsies were obtained under the prevailing environment conditions (i.e., in hypoxia during HBR and HAmb and in normoxia during NBR). The procedure was performed in a supine position on the non-dominant leg with the sample obtained from the superficial mid–portion of the vastus lateralis. Muscle samples were taken by a medical doctor experienced with the use of Rongeur biopsy forceps using standard procedures. Obtained samples were immediately weighed, sectioned, and snap frozen in liquid nitrogen. All samples were subsequently kept in -80°C freezers. For final analysis, samples were ATPase stained in order to determine fiber type composition.

### Data Analysis

Linear mixed effect (LME) models with time and condition (either NBR, HBR, or HAmb) as fixed effects and participants as random effect, were used to assess differences in changes from baseline across conditions. One-sample *t*-tests were used to assess differences in muscle biopsy sample analyses to assess differences from baseline. Statistical analyses were performed for the changes obtained at the end of bed rest. In order to test the first hypothesis, namely a difference between NBR and HBR, LME models were constructed with condition (NBR and HBR) as fixed effect and subject as random effect. Variances could differ between conditions, and box-cox transformation was performed where required by distorted q-q plots or heteroscedasticity. The secondary hypothesis was tested via a 1-sided *t*-test of changes from baseline in HAmb data. When no difference between NBR and HBR was found (β = 0.2), the two conditions were lumped together and a 1-sided *t*-test was performed against 0. The level of statistical significance was set *a priori* at 0.05. Data are presented as means ± standard deviations (SD) and means ± standard errors (SE) only where specified. All statistical analyses were performed using the R-environment (version 3.1.1)^1^.

## Results

### General Adaptation to Bed Rest and Hypoxia

Participants underwent all three interventions without any significant unwanted events except for one participant who, upon acute exposure to 4000 m simulated altitude during the HBR intervention, experienced severe hypoxemia-related dizziness accompanied by mild nausea. He was consequently moved to a different room with a lower simulated altitude (approx. 3000 m). For this individual, the simulated altitude was thereafter increased by 500 m for the next 2 days. The same gradual increase was subsequently used during his HAmb intervention. Due to personal reasons two individuals did not return for the third campaign. A third participant did not finish the third campaign due to medical reasons unrelated to the study. Of these three individuals, data from only two campaigns was used for subsequent analysis. While the average SpO_2_ values were 97 ± 2% during NBR, they were significantly lower during both hypoxic interventions (HBR = 88 ± 2%; HAmb 88 ± 1%; *P* < 0.001). SpO_2_ values gradually and significantly increased during both hypoxic interventions with the average values of 90 ± 2% and 89 ± 2% during the last day of the HBR and HAmb intervention, respectively (*P* < 0.05). The LLS scores obtained during the first 5 days of each intervention suggest that AMS was observed in five participants during the HBR and three participants during the HAmb intervention. The average HR responses during the HAmb exercise sessions were in line with the targeted values (124 ± 9 beats⋅min^-1^).

### Dietary Intakes

As reported previously (Debevec et al., 2014a; Debevec et al., 2016), the actual energy intakes (NBR = 2027 ± 188; HBR = 2018 ± 202; HAmb = 2197 ± 193) were significantly lower than targeted (NBR and HBR = 2139 ± 193; HAmb = 2558 ± 226 kcal; *P* < 0.01) in all campaigns. Nevertheless, percentages of daily intakes of protein (16 ± 1, 17 ± 1, 17 ± 1), fat (30 ± 1, 29 ± 1, 30 ± 1) and carbohydrates (54 ± 1, 54 ± 1, 53 ± 1) were comparable across NBR, HBR, and HAmb, respectively. In addition, average water (3.5 ± 1, 3.6 ± 1, 3.6 ± 1 L) and sodium (2374 ± 335, 2357 ± 321, 2630 ± 360 mg) intakes were similar between NBR, HBR, and HAmb.

### Muscle Cross-Sectional Area

**FIGURE 2:**
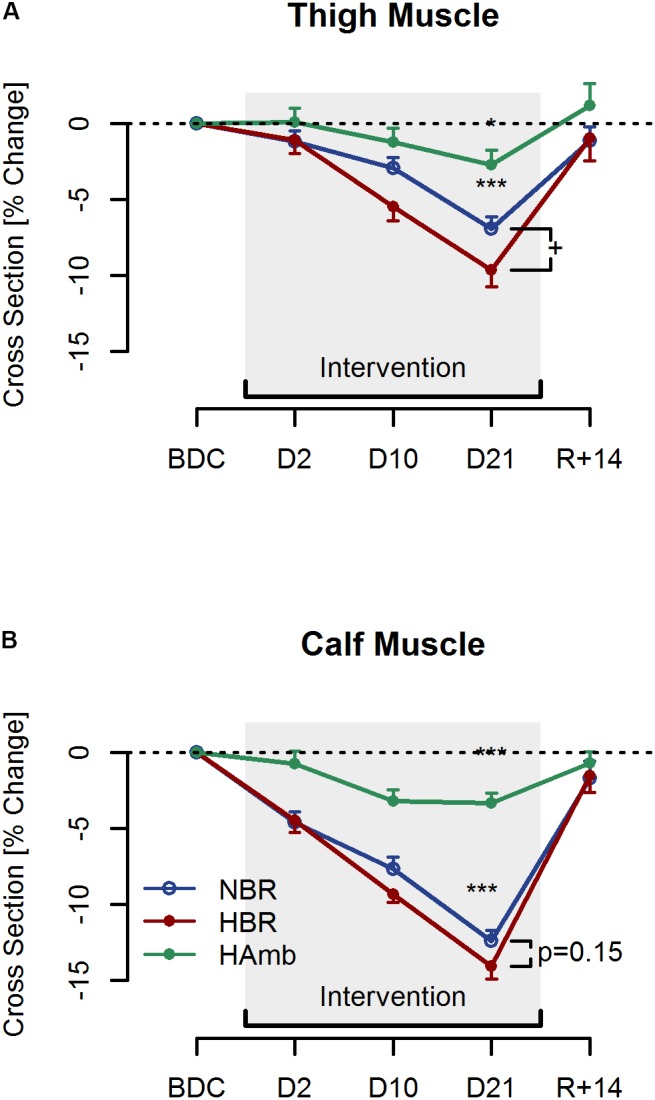
Thigh **(A)** and Calf **(B)** muscle cross-sectional area changes after two (D2), ten (D10), and twenty-one (D21) days of normoxic bed rest (NBR; blue), hypoxic bed rest (HBR; red) and hypoxic ambulation (HAmb; green) interventions, as well as 14 days post (R+14). Data are presented as Means ± SE. ^∗^(*p* < 0.05) and ^∗∗∗^(*p* < 0.001) denote significant reductions compared to baseline. + (*p* < 0.05) denotes significant difference between the two bed rest interventions.

Thigh and calf muscle cross-sections at baseline of the NBR, HBR, and HAmb interventions are given in **Table 1**. While no baseline differences were found for thigh muscle size (*P* = 0.50), a trend for calf muscle size difference was noted (*P* = 0.066). Changes in thigh and calf muscle size across all three interventions are presented in **Figure 2**. No significant differences in both thigh and calf muscle size were observed following only 2 days of each intervention (*P* > 0.05; **Figure 2**). After 10 days of both bed rest interventions, a comparable reduction in thigh size was observed (NBR = -2.6%, SE 0.6%; HBR = -5.4%, SE 0.8%). After 21 days of NBR, thigh muscle size was reduced by -6.9% (SE 0.8%; *P* < 0.001; **Figure 2**) and this reduction was further aggravated to -9.7% (SE 1.2%) in HBR (*P* = 0.027). Thigh muscle size was reduced less following 21 days of HAmb (-2.7%, SE 1.1%, *P* = 0.017). The significant 21-day bed rest-induced muscle wasting in the calf (NBR = -13.3%, SE 1.1%) was not further enhanced by hypoxic conditions (HBR = -14.9%, SE 1.3%, *P* = 0.47). Ambulatory hypoxia resulted in slight, but statistically significant muscle wasting in the calf (-3.3%, SE 0.7%, *P* < 0.001).

**Table 1:** Thigh and calf muscle cross-sectional areas, as assessed by pQCT, and fiber type composition of the vastus lateralis muscle, as assessed from biopsy material. Data are presented as means ± SD.

	NBR	HBR	HAmb
Thigh muscle CSA (cm^2^)	123.1 (20.7)	121.1 (18.8)	124.3 (23.3)
Calf muscle CSA (cm^2^)	80.2 (15.7)	79.4 (13.9)	81.4 (14.5)
Vastus lateralis type 1 (%)	37.9 (8.6)	37.2 (7.5)	39.0 (8.1)
Vastus lateralis type 2a (%)	51.9 (15.4)	40.8 (15.4)	45.6 (10.8)
Vastus lateralis type 2× (%)	10.2 (10.8)	22.0 (13.5)	15.4 (8.9)

### Muscle Fiber Type

**FIGURE 3:**
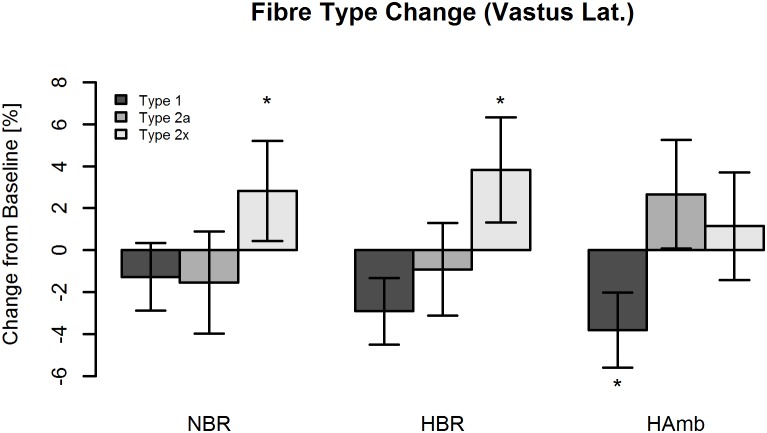
Type 1, type 2a, and type 2× fiber changes within the vastus lateralis muscle following normoxic bed rest (NBR), hypoxic bed rest (HBR), and hypoxic ambulation (HAmb) interventions. Data are presented as means ± SE. ^∗^(*p* < 0.05) denotes significant difference compared to baseline.

Fiber type distributions at baseline of NBR, HBR, and HAmb are given in **Table 1**. There were no differences between conditions for the percentage of type 1 fibers (*P* = 0.89), but trends for type 2a and type 2× fibers (*P* = 0.054 and *P* = 0.077, respectively). **Figure 3** depicts the changes in fiber type percentages of vastus lateralis muscle following all three interventions. A significant increase in type 2× fiber percentage was observed at the end of both, normoxic (NBR = +3.1%, SE 2.6%, *P* < 0.05) and hypoxic (HBR = +3.9%, SE 2.7%, *P* < 0.05) bed rest interventions. The observed increase in type 2× percentage was comparable between the two conditions (*P* = 0.76; **Figure 3**). Moreover, a trend was found for a decrease in type 1 fibers (*P* = 0.057) when lumping HBR and NBR data together, without any difference between these two conditions (*P* = 0.45). By contrast, no effect of bed rest (HBR, NBR, or both lumped together) was observed for type 2a fibers (*P* > 0.40), and likewise no difference between conditions (*P* = 0.26). For HAmb, a significant reduction in type 1 fiber percentage was noted (-3.6%, SE 2.3%, *P* < 0.05), but there were no changes in the percentage of type 2a or type 2× fibers (*P* = 0.26 and *P* = 0.63, respectively).

## Discussion

This work sought to experimentally elucidate potential effects of systemic environmental hypoxia on inactivity-related thigh and calf muscle wasting. Our findings demonstrate accentuated thigh muscle wasting and fiber type shift induced by a combination of hypoxic exposure and bed rest-induced inactivity. Importantly, hypoxia-related exacerbation of muscle wasting was observed under precisely controlled and standardized environmental, dietary and activity conditions. The fact that these effects were quite readily developing in young and healthy men implies that aging and/or presence of chronic diseases is not a prerequisite for hypoxia to aggravate muscle wasting.

### Independent Effects of Inactivity

As mentioned previously numerous studies to-date have demonstrated significant muscle mass wasting in the lower limbs following whole body inactivity of various duration. This inactivity-related muscle wasting is thought to be predominantly underlined by disruption of protein synthesis and breakdown balance (Gamrin et al., 1998). Expectedly, data obtained during the NBR intervention are in line with previous bed rest studies of similar duration (Kawakami et al., 2000, 2001; Akima et al., 2001; Kubo et al., 2004). Interestingly, analysis of the obtained biopsy samples from the NBR intervention (Rullman et al., 2016) suggests that miRNA expression did not drive the observed muscle phenotype modifications with inactivity. As demonstrated previously, a significant individual variability in muscle atrophy responses was found in the present study (Narici and de Boer, 2011). In particular, individual bed rest-related responses varied from 2 to 16% reduction for the thigh and 7 to 18% reduction for the calf. The observed difference between the thigh and the calf further has been repeatedly found in earlier bed rest studies. It seems straightforward to ascribe this difference to the muscle group’s importance for weight-bearing and ambulation (Berg et al., 2007), but a detailed analysis of the various muscle groups does not support this notion (Belavy et al., 2009). Finally, the muscle phenotype changes derived from muscle biopsies clearly show an inactivity-related increase in type 2× fibers confirming the previously reported shift from oxidative to glycolytic fiber type (Brooks and Myburgh, 2014; Demangel et al., 2017).

### Additive Effects of Hypoxia

Previous work on hypoxia-related muscle tissue structural changes implies that the observed muscle wasting is mostly driven by mTOR-dependent protein synthesis inhibition and transient proteolysis activation (Favier et al., 2015). Regardless of the discrepancies between study outcomes (Boutellier et al., 1983; Hoppeler et al., 1990; MacDougall et al., 1991; Green et al., 2000; Lundby et al., 2004; D’Hulst et al., 2016; Pasiakos et al., 2017) hypoxia does seem to modulate muscle mass regulation in a dose-dependent manner (Bigard et al., 1996; Favier et al., 2010). Additive effects of hypoxic exposure were clearly notable in the present study with significantly accentuated muscle wasting in the thigh following HBR as compared to NBR. Our results thus indicate that 21 days of exposure to simulated altitude of 4000 m can already result in significantly altered muscle modulation. This is especially interesting with regards to the hypoxic dose required for a measurable effect, since previous studies demonstrating positive correlation between exposure to hypoxia and muscle wasting in humans (Boutellier et al., 1983; Hoppeler et al., 1990; MacDougall et al., 1991) employed higher altitude exposures (i.e., >5000 m). Furthermore, a significantly higher overall hypoxic dose (e.g., exposure to ≥5000 m for ≥4 weeks) has recently been suggested as threshold for hypoxia-induced muscle wasting (D’Hulst and Deldicque, 2017). Other than in the thigh, no additive effect of hypoxia was noted in the calf. A possible explanation could be the potentially greater role of bed rest-induced fluid shifts on the calf than on the thigh. In addition, greater power and overall muscle mass of the thigh as compared to the calf may also result in greater oxygenation dependence of the thigh. Of note, comparable muscle fiber type shifts in the vastus lateralis were observed following NBR and HBR (i.e., significant increases in type 2× fiber percentage), suggesting the employed hypoxic dose did not significantly influence fiber type remodeling. The fact that vastus lateralis muscles of young, healthy males are known to comprise a relatively high proportion of the type 1 fibers (Staron et al., 2000) could, at least partly, explain the lack of observed differences since hypoxia has been shown to predominantly affect type 2 fibers (de Theije et al., 2015). Collectively, data obtained during the HBR campaign clearly indicate that the combination of both, hypoxemia and inactivity, seems particularly harmful for thigh muscle tissue.

### Effects of Ambulatory Hypoxia

While the purpose of the third arm of the project (i.e., HAmb intervention) was to elucidate the independent effects of hypoxia, the lack of a normoxic ambulatory control group, which could not be implemented due to logistical and financial constraints, limits the interpretation of the obtained data. In contrast to our initial hypothesis, the HAmb intervention provoked a slight, albeit significant, reduction in both, thigh and calf muscle size. Similar to the thigh and calf cross-sectional area, discussed here, our previously published analysis of DEXA-derived body composition data also indicated that all interventions provoked reductions in whole-body fat-free mass (Debevec et al., 2014a). One could speculate that this overall fat-free mass reduction might be a consequence of lower than targeted energy intakes noted in all three interventions, even though the subjective appetite did not seem to be affected (Debevec et al., 2016). The fact that the intensity of exercise performed during the HAmb was rather low might have contributed to the rather surprising muscle mass reductions. Indeed, muscle protein synthesis, one of the key muscle tissue adaptive activity responses, is known to be almost unaffected by low intensity aerobic (Short et al., 2004) or resistance (Atherton and Smith, 2012) exercise. In addition, the previously reported suppression of exercise-related protein synthesis in response to both, acute (Etheridge et al., 2011) and chronic (Narici and Kayser, 1995) hypoxia might have influenced the observed outcomes. As discussed previously (Debevec et al., 2014a,b), confinement *per se* might have played a role by limiting habitual activity levels even though participants were strongly encouraged to maintain comparable levels to those encountered in their daily lives. On the other hand, significantly lower than targeted energy intakes during the HAmb, could also have contributed to the observed thigh and calf muscle mass reduction. While a significant decrease in type 1 fiber percentage of the vastus lateralis following HAmb was rather surprising, similar reductions were previously reported in active individuals undergoing lab-based (MacDougall et al., 1991) or terrestrial (Mizuno et al., 2008) hypoxia/altitude exposures.

### Clinical Implications

While we acknowledge that the observed effects in healthy individuals may not necessarily be extrapolated to clinical populations, the present study provides the first direct insight into the negative effects of hypoxia *per se* on inactivity-related muscle wasting in humans. Besides the two investigated factors (i.e., hypoxia and (in)activity), COPD or heart failure patients also suffer from numerous other influential disease-related pathophysiological abnormalities. Importantly, in the present study, the additive detrimental effects of hypoxia were readily observed in the absence of other potential confounding factors such as nutritional deficits and under precisely controlled experimental conditions. While earlier studies suggest (Boutellier et al., 1983; Hoppeler et al., 1990; MacDougall et al., 1991) that the dose of hypoxia needed for observable muscle wasting has to be much higher (i.e., terrestrial exposure >5000 m), it seems that even lower hypoxia levels, as employed in the present study, can exert a negative effect on thigh muscle mass. This finding is, in turn, especially pertinent for COPD patients who are known to exhibit persistent systemic hypoxia with the arterial O_2_ concentrations corresponding to the levels measured in healthy individuals at altitudes between 1500 and 3000 m (Wagner, 2008). Inactivity-related fiber-type shifting to type 2 fibers is also a potentially important observation of this study pertinent to COPD patients. Especially, since their muscles are enriched with type 2 fibers compared to their healthy counterparts (Gosker et al., 2002) and moreover, given that fiber shifting toward glycolytic-type fibers has been related to increased fatigability, reduced endurance and reduced oxidative capacity of the COPD patients lower limb muscles (Kim et al., 2008). Based on the above and given the previously reported correlations between muscle wasting and mortality in patient populations (Marquis et al., 2002; Swallow et al., 2007; Patel et al., 2014), strategies to prevent and/or alleviate both, inactivity (e.g., exercise interventions) and systemic hypoxemia are clearly warranted. Indeed, present work provides a starting point for further well-controlled, long-term, prospective investigations into potential benefits of inactivity and hypoxia mitigation strategies in various clinical populations.

### Limitations and Methodological Considerations

Whereas the present study is the first strictly controlled investigation into the additive effects of hypoxia on inactivity-related muscle wasting, there are a few considerations that we would like to bring to the readers’ attention. The main one relates to the employed inactivity duration and hypoxic dose. As mentioned previously, both inactivity and hypoxia are known to exert a dose-dependent effect of skeletal muscle mass modulation. While muscle-related effects of similar duration bed-rest are clearly established and were also confirmed in the present study, effects of the hypoxic dose (i.e., intensity and duration) are less clear. It was recently hypothesized, that for a robust muscle wasting response at least the intensity (i.e., the reduction of P_i_O_2_) should be higher (D’Hulst and Deldicque, 2017). Nevertheless, our data show that even a lower overall hypoxic dose can detrimentally influence muscle tissue when combined with inactivity. In addition, potential effects of confinement *per se*, as experienced by the participants within the facility need to be acknowledged. Prolonged confinement has previously been shown to relevantly influence nutritional status, appetite and body mass modulation (Westerterp et al., 1988; Custaud et al., 2004) and might have thus, confounded our results. While this work has been motivated by the interest to study clinical aspects of combined hypoxia and unloading, the PlanHab project initially aimed to determine whether concomitant exposure to these two factors, as envisaged during future planetary habitation during prospective deep space exploration, will negatively influence operational abilities of future astronauts. In this regard, the present findings suggest that exposure to hypoxia during periods of unloading (i.e., weightlessness or microgravity), might prove detrimental for muscle tissue of the thigh and should thus be taken into account when planning muscle wasting mitigation strategies during future deep-space explorations. Finally, one should bear in mind that a cross-over design has not only strengths (foremost reduction of inter-subject variability), but also the dilemma of defining the washout period between interventions. On basis of recovery of muscle atrophy after bed rest (Rittweger and Felsenberg, 2009), we had defined a period of 4 months, which was more than five times longer than the 21-day intervention phase. Baseline values were very comparable for thigh muscle size and type 1 fiber percentage (**Table 1**). A statistical trend indicated a possible baseline-divergence of calf muscle size (which was small compared to experimental effects) and type 2a/2× fiber percentage (which was substantial in comparison to experimental effects). It remains unclear whether this possible baseline divergence results from a combined seasonal and carry-over effect, and it is also not clear whether a longer wash-out would have been helpful. Based on the relative stability of baseline values, we feel that the 4-month duration was an acceptable compromise between long enough wash-out and the risk of subject drop-out.

## Conclusion

In summary, the present findings suggest that prolonged exposure to combined hypoxemia and lack of activity might be particularly harmful for muscle tissue. Importantly, aging does not seem to be a prerequisite for hypoxia-related aggravation of muscle wasting, since these effects were readily observed in young, healthy individuals. This does not only seem to be highly relevant for and supportive of exercise interventions in clinical populations, but also for critical care medicine when trying to prevent muscle wasting in individuals subjected to systemic and prolonged unloading and hypoxemia (Puthucheary et al., 2013).

## Author Contributions

OE, IM, and JR contributed conception and design of the study. TD, BG, OE, IM, and JR conducted the experiments. TD, UM, and JR organized the database. JR performed the statistical analysis. TD and JR drafted the manuscript. All authors contributed to manuscript revision, read, and approved the submitted version.

## Conflict of Interest Statement

The authors declare that the research was conducted in the absence of any commercial or financial relationships that could be construed as a potential conflict of interest. The reviewer TD and the handling Editor declared their shared affiliation.

## Footnotes

1 www.r-project.org
